# Normality Index of Ventricular Contraction Based on a Statistical Model from FADS

**DOI:** 10.1155/2013/617604

**Published:** 2013-03-24

**Authors:** Luis Jiménez-Ángeles, Raquel Valdés-Cristerna, Enrique Vallejo, David Bialostozky, Verónica Medina-Bañuelos

**Affiliations:** ^1^Nuclear Cardiology Department, Instituto Nacional de Cardiología “Ignacio Chávez”, Juan Badiano No. 1 Colonia Seccion XVI, Tlalpan, 14080 Mexico City, DF, Mexico; ^2^Neuroimaging Laboratory, Electrical Engineering Department, Universidad Autónoma Metropolitana-Iztapalapa, San Rafael Atlixco No. 186 Colonia Vicentina, Iztapalapa, 09340 Mexico City, DF, Mexico

## Abstract

Radionuclide-based imaging is an alternative to evaluate ventricular function and synchrony and may be used as a tool for the identification of patients that could benefit from cardiac resynchronization therapy (CRT). In a previous work, we used Factor Analysis of Dynamic Structures (FADS) to analyze the contribution and spatial distribution of the 3 most significant factors (3-MSF) present in a dynamic series of equilibrium radionuclide angiography images. In this work, a probability density function model of the 3-MSF extracted from FADS for a control group is presented; also an index, based on the likelihood between the control group's contraction model and a sample of normal subjects is proposed. This normality index was compared with those computed for two cardiopathic populations, satisfying the clinical criteria to be considered as candidates for a CRT. The proposed normality index provides a measure, consistent with the phase analysis currently used in clinical environment, sensitive enough to show contraction differences between normal and abnormal groups, which suggests that it can be related to the degree of severity in the ventricular contraction dyssynchrony, and therefore shows promise as a follow-up procedure for patients under CRT.

## 1. Introduction

Heart failure (HF) is defined as a complex clinical syndrome that can result from any structural or functional cardiac disorder and that impairs the ability of the ventricle to fill or eject blood [1]. According to a 44-year followup of the National Heart, Lung, and Blood Institute's Framingham Heart Study, approximately 5.7 million patients have an HF diagnosis in the United States. After HF is diagnosed the survival rate is lower in men than in women, less than 15 percent of women survive more than 8–12 years and the one-year mortality rate reaches 20% [2].

Ventricular dyssynchrony has also been associated with increased mortality in HF patients [3, 4]. Dyssynchronous contraction can be palliated by electrically activating in a synchronized form the right and left ventricles with a multisite pacemaker device. This kind of treatment is called cardiac resynchronization therapy (CRT). Several clinical studies have shown that CRT contributes to an increase in the life expectancy of subjects diagnosed with cardiac failure, specifically of the type where the left ventricle ejection fraction is under 35% or classified in levels III or IV, according to the New York Heart Association [5–7] criteria. In a meta-analysis of several CRT trials, evidence showed that HF hospitalizations were reduced by 32% and that all-cause mortality decreased by 25% after approximately 3 months of therapy [8]. In a randomized controlled trial comparing optimal medical therapy alone with optimal medical therapy plus CRT (without a defibrillator), CRT significantly reduced the combined risk of death by any cause and decreased the unplanned hospital admission for a major cardiovascular event by 37% [9].

However, 20% to 30% of patients having HF do not benefit from resynchronization therapy, probably due to several causes [10].

- The established criteria to select candidates for CRT are limited.
- The stimulation leads are not properly placed.
- There is excessive fibrous tissue at the stimulus location.

This has led to the definition of several indexes, extracted from imaging modalities, to measure the cavities mechanical contraction, and that allow the proper identification of candidates to undergo CRT [11]. The quantification of peak systolic velocity and myocardial deformation from echocardiographic images has been proposed as representative indexes to evaluate ventricular dyssynchrony. However, studies carried out in multiple health centers have shown that it has a very low sensitivity to discriminate subjects who respond to CRT from those who do not [12]. Recent studies have reported indices, extracted from MRI, which can contribute to the solution of this problem, but the use of this imaging modality is restricted, depending on the type of resynchronization device that has been implanted [11, 13–16].

**Figure 1:**
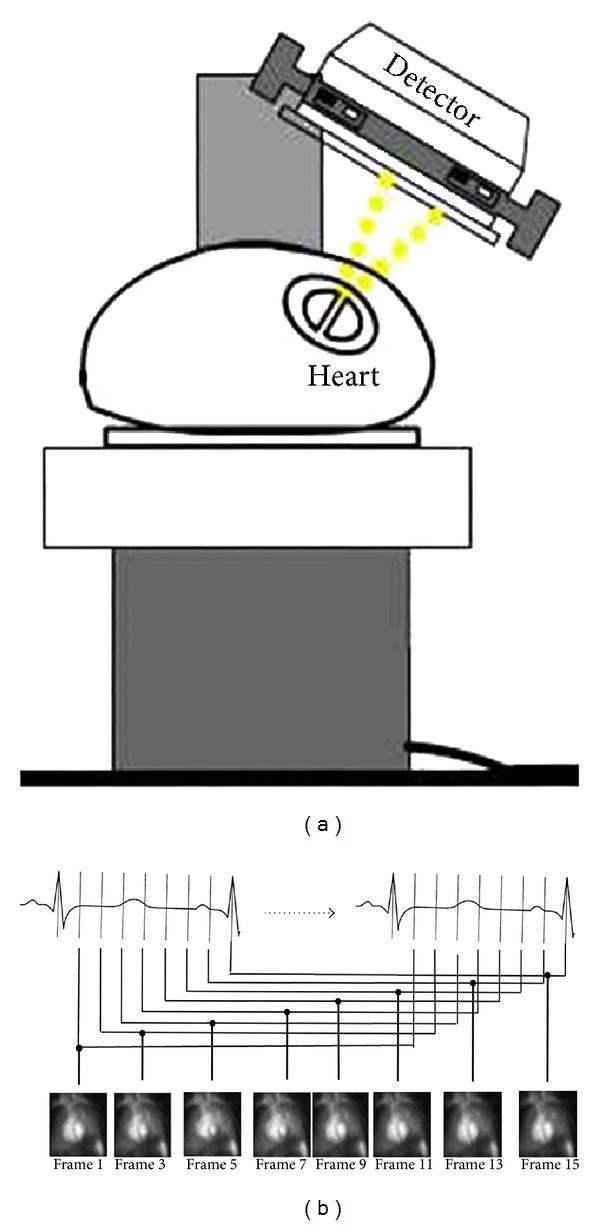
Schematic ERNA image acquisition. (a) Detector in the left anterior oblique (LAO) position to visualize the best RV and LV definition. (b) Several EKG-gated temporal frames corresponding to different phases of cardiac cycle are acquired in the LAO position. The ERNA images are obtained from summation of individual frames.

Radionuclide-based imaging is another alternative to evaluate ventricular contraction synchrony [17]. The equilibrium radionuclide angiography (ERNA) is a set of images that represent the spatial distribution of a radiotracer and relates pixel's intensity to ventricular volume. The general setup for ERNA image acquisition (see Figure 1) consists in locating the detector of the gamma camera in the left anterior oblique view (LAO) with patient at rest in supine position after the injection of red blood cells marked with Tc-99m. Synchronized acquisition of images with the electrocardiogram (EKG) allows the accumulation of radioactivity in several R-R intervals, to construct the image set that represents a specific instant of the cardiac cycle [18, 19].

**Figure 2:**
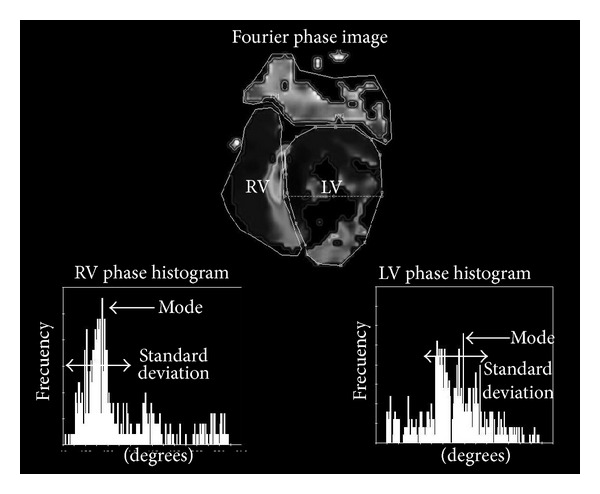
Parametric Fourier phase image and the phase image histogram for both ventricles (RV and LV). Indices like mean, standard deviation, and mode, computed from the statistical distribution of the phase angles, have been proposed to evaluate contraction abnormality patterns.

ERNA images are processed by adjusting the first harmonic component of the Fourier Transform (FT) of each pixel's temporal intensity evolution (time-activity curve (TAC)). From these components, phase angles, which are representative of the TAC behavior, are extracted and a map (phase image) of the ventricular contraction sequence is constructed [20]. Several indices, taken from the statistical distribution of the phase angles, have been proposed to detect contraction abnormality patterns [21–23].  Figure 2 shows an example of the phase image corresponding to an abnormal contraction pattern (with intraventricular and interventricular dyssynchrony), together with the right (RV) and left (LV) ventricle histograms. The modes and standard deviations of these distributions are measured and used as clinical indices to identify abnormalities.

The standard deviation of the pixels' phase angles measured in each ventricular Region of Interest (ROI) represents intraventricular dyssynchrony and the difference between the means of the phase angles of both ventricular ROI represents interventricular dyssynchrony [24]. Several studies have reported an improvement of interventricular and intraventricular dyssynchrony after CRT, using the Fourier phase analysis of ERNA images [25–29]. Dauphin et al. [30] showed that interventricular dyssynchrony was identified as an independent predictive factor of good clinical response with a practical cut-off value of 25.5°, a sensitivity of 91.4%, and a specificity of 84.4%. However, Fourier phase analysis based only on one FT harmonic has its limitations, since it assumes periodic TACs and a smooth transition between the first and last frame of the dynamic images series. These drawbacks are more prominent in the regions with severe contraction pattern abnormalities. 

Factor Analysis of Dynamic Structures (FADS) has also been proposed as a valuable tool to detect abnormalities in ventricular cavities' movement [31, 32]. It is applied to ERNA images to extract those TACs associated to the physiological behavior of a specific region and assumes that there are pixel clusters with the same temporal evolution which define their morphology. Therefore, FADS determines the TACs (coefficients) of pixel groups with the same behavior, in addition to their geometry and spatial location (factors) [33, 34]. In a previous work carried out by our group, we analyzed the contribution and spatial distribution of the most significant factors present in a dynamic series of ERNA images and we proposed an alternative method to reconstruct the phase image. In [35], we reported that more than 90% of the information contained in an image series is represented by the three most significant factors (3-MSF) and that the third factor increases considerably whenever an abnormality of the contraction pattern is observed. Also, a detailed analysis of the scatter plots of the 3-MSF showed the importance of the third factor to adequately separate regions having an abnormal contraction pattern. Therefore, the need to propose an index to quantify contraction abnormality, using the representative information extracted from dynamic image series, becomes evident.

In this work, a probability density function model of the 3-MSF, extracted from FADS for a control group, is presented; also a reference normality index, based on the likelihood between the control group's contraction model and a sample of normal subjects, is proposed. The index was then statistically compared with those computed for two populations of patients satisfying the clinical criteria to be considered as candidates for a CRT: a group with complete left bundle branch block (LBBB) and a group with dilated cardiomyopathy (DCM). 

The paper is structured as follows: in the Methodology section we describe the proposed model to characterize a normal contraction pattern (Sections 2.1 and 2.2); the defined index to quantify the degree of normality with respect to a reference population (Section 2.3); the populations considered to test the proposed index (Sections 2.4 and 2.5) as well as the statistics employed (Section 2.6). The Results section describes the findings of the proposed normality index tested in different cardiopathies and compared to the clinical standard provided by Fourier phase analysis. These are analyzed and interpreted in the Discussion section, to finally conclude at the corresponding section.

## 2. Materials and Methods

### 2.1. Factor Analysis of Dynamic Structures (FADS)

**Figure 3:**
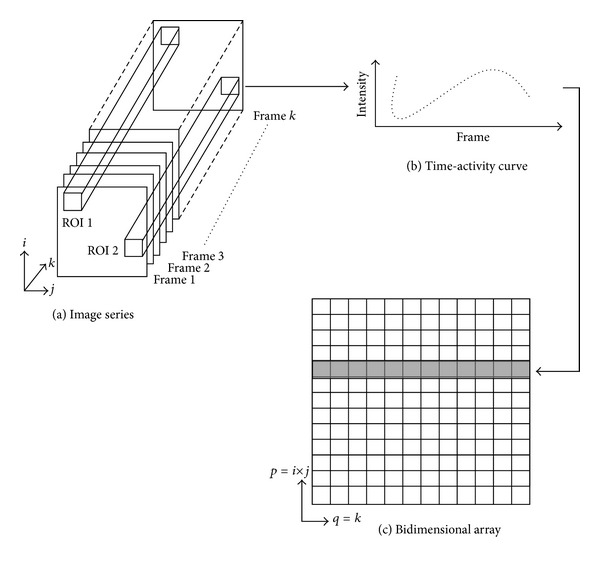
(a) ERNA study consisting of a *k*-images series, with frames having *i* × *j* pixels. (b) Time-activity curve extracted from a particular Region of Interest (ROI 1). (c) Bi-dimensional array constructed from the image series.

Let **X**_TAC_(*p*, *q*) = **X**[(*i*, *j*), *k*] be a bidimensional array (Figure 3(c)), whose indices represent the (*i*, *j*)th pixel value of the *k*th frame of the acquired image series. Each frame size is *M* × *M* pixels (*p* = (*i* − 1) × *M* + *j*, *q* = *k*) as shown in Figure 3(a). **X**_TAC_(*p*, *q*) represents the time-series generated for each pixel on the image set, known as time-activity curves (TAC) (Figure 3(b)).

Let **Q** be a linear transformation that decorrelates the ERNA image set (**X**_TAC_), so that

(1) $$\begin{array}{c} F\;=\;X_{m}Q,\\ Q\;=\;VD, \end{array}$$

where **F** are the factors of **X**_TAC_(*p*, *q*) (Figure 3(c)), **X**_*m*_ is **X**_TAC_(*p*, *q*) with the mean-value removed; **V** is the eigenvector set of the autocorrelation matrix of **X**_*m*_ and **D** is the scaled diagonal matrix of the eigenvalues set of the autocorrelation matrix of **X**_*m*_. The contribution of each factor is determined by the corresponding eigenvalue magnitude.

### 2.2. Normal Contraction Pattern Model

**Figure 4:**
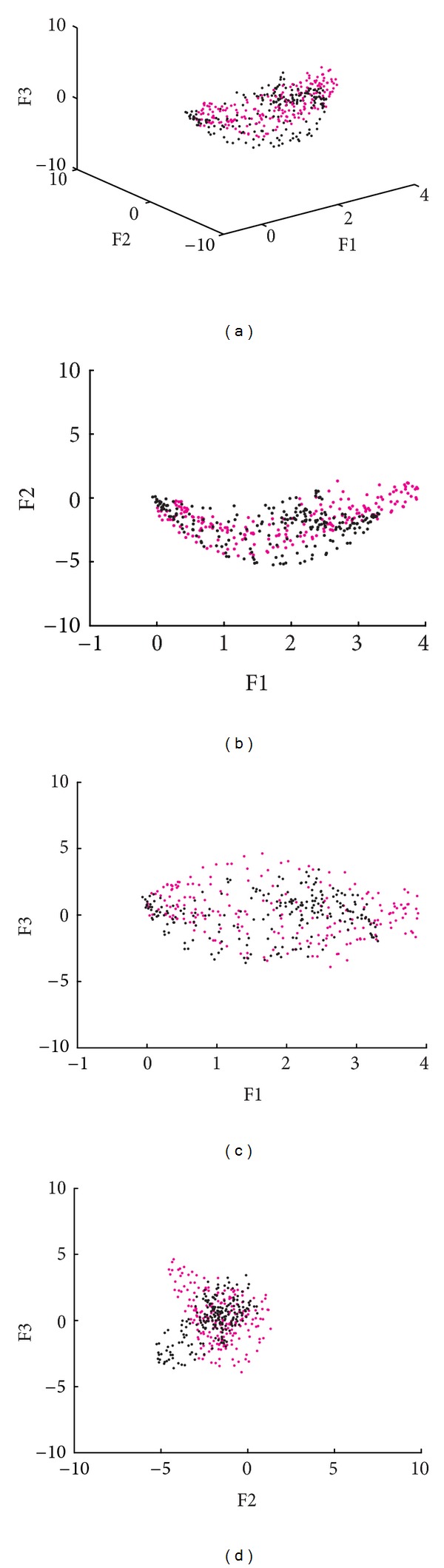
Scatter plot of the 3-MSF (F1, F2, and F3) calculated for a subject with a normal contraction pattern (right ventricle in magenta, left ventricle in black).

The three most significant factors (3-MSF) of the ERNA studies, obtained for a population of normal subjects, were analyzed; the spatial distribution of those factors can be observed in Figure 4. Every point in the factorial 3D space is associated to the projection of a given pixel in the ERNA image, into each of the main eigenvectors **V**.

The probability density function (PDF) of those factors was modeled by a linear combination of *R* Gaussian density functions (Gaussian mixture), defined by the following expression [36]:

(2) $$\begin{array}{c} p\left(f\right)=\sum_{r=1}^{R}w_{r}N\left(f\;|\;\mu_{r},\Sigma_{r}\right), \end{array}$$

where *f* is observed variable, *w*_*r*_ is relative weight of the *r*th gaussian function of the mixture, and *N*(*μ*_*r*_, Σ_*r*_) is the multivariate Gaussian PDF with *μ*_*r*_ and Σ_*r*_ parameters

(3) $$\begin{array}{c} \sum_{r=1}^{R}w_{r}=1,\;0\leq w_{r}\leq 1. \end{array}$$

To estimate the mixture parameters {*w*_*r*_,   *μ*_*r*_ *y* Σ_*r*_} for *r* = 1,…, *R*, the expectation maximization algorithm, that maximizes the mixture model likelihood, was used [37].

Six data groups were assembled, with 10 subjects each, randomly selected from a total of 23 control subjects, following a standard bootstrap resampling procedure. The PDF of the 3-MSF was modeled for each group, using the procedure described above. The number of components of the Gaussian mixture was determined considering the bayes information criterion (BIC) [38, 39]. The models' parameters, BIC, and likelihood were calculated using the R package (R Foundation, http://www.r-project.org/) [40].

### 2.3. Normality Index

Considering that the likelihood estimated on a data sample (*f*_*s*_) represents the probability that those observations are well described by the assumed model (Gaussian mixture with *w*, *μ*, and Σ parameters), in this work we propose a normality index based on this probability. Assuming statistical independence between observations, the average log-likelihood of a sample set with respect to the reference (healthy) population can be defined as a comparative index (*I*_*N*_) of a normal contraction pattern as follows:

(4) $$\begin{array}{c} I_{N}=\frac{1}{||S||}\sum_{s\in S}^{}\log\left(\sum_{r=1}^{R}w_{r}N\left(f_{s}\;|\;\mu_{r},\Sigma_{r}\right)\right), \end{array}$$

where *S* is the observations' set, that in our case corresponds to the ventricular region TACs for each subject.

The normality index (*I*_*N*_) for a group of eight normal subjects (not considered for the mixture parameters estimation) was measured and statistically compared with the indices obtained for LBBB and DCM subjects.

### 2.4. Studied Populations

Three subject groups were considered in this study: 15 subjects with LBBB; 13 patients with DCM and 31 normal subjects (23 as a control population for the training stage and 8 to define the normality index); all individuals gave their informed consent to participate in the study. The specific characteristics for these populations are shown in Table 1.

LBBB occurs whenever the electric impulse traveling from auricles to ventricles is interrupted, thus causing a QRS complex duration longer than 0.12 s. This delay provokes interventricular contraction asynchrony which can progress to eventually become cardiac insufficiency [41]. The LBBB studied population consisted of 15 asymptomatic subjects (8 males, 7 females), having a left ventricle ejection fraction (EF) greater than 45%, as determined by ERNA according to the New York Heart Association (NYHA) criteria [42]; the subjects did not present cardiovascular symptoms and did not have a previous history of myocardial infarct and/or cardiac insufficiency. 

Subjects with idiopathic DCM and cardiac insufficiency present left ventricle (LV) or right ventricle (RV) dilatation of unknown causes, inter- and intraventricular abnormal contractility; they must reunite all of the criteria to be considered as CRT candidates [41–44]. The DCM population consisted of 13 subjects, with EF of 22.2 ± 6.7%, as determined by ERNA, and with an average QRS duration of 0.160 ± 0.26 s; they also presented a class III or IV cardiac insufficiency, according to the NYHA [42].

The control population consisted of 23 volunteers (18 males, 5 females) having an EF of 60 ± 5.84%; with a low probability of coronary arterial disease and without a history of myocardial acute infarct. This group presented an EKG without abnormality and their cardiac function was considered normal, after a thorough clinical evaluation.

### 2.5. ERNA Images Acquisition

The same *General Electric* millenium MPR/MPS gamma camera was used for all ERNA image acquisition. It has a single head with 64 photomultiplier tubes and it is equipped with a low energy, high resolution parallel-hole collimator; the calibration of the energy peak was centered at 140 KeV and the detector's uniformity was guaranteed at less than 5% [45]. Images were digitized at a 64 × 64 pixels resolution and 1.33 zoom factors.

Erythrocytes were marked applying an *in vivo/in vitro* modified technique with 740 to 925 MBq of Tc-99m, using an UltraTag Kit [46, 47]. EKG was continuously monitored to synchronize images acquisition with the R wave. To eliminate ventricular extrasystoles during acquisition, a beat acceptance window was defined at ±20% of the average heart rate. Images were taken in an anterior left oblique projection, in order to simultaneously attain the best definition of left and right ventricles. A total of 16 frames were obtained with a density of 300 Kcounts per frame. 

For each subject, an image corresponding to the end of diastole was selected and manually segmented by an expert, to define the ventricular area. This segmentation defines a mask that is used to automatically extract the ventricular regions from the other frames.

### 2.6. Statistical Analysis

The normality indices are expressed as the mean value ± standard deviation. Indices measured for the normal group were independently compared to those obtained for LBBB and DCM populations, using a *t*-test for independent samples and considering *P* ≤ 0.01. The SPSS version 10.0 software was used for all statistical analyses.

### 2.7. Summary

**Figure 5:**
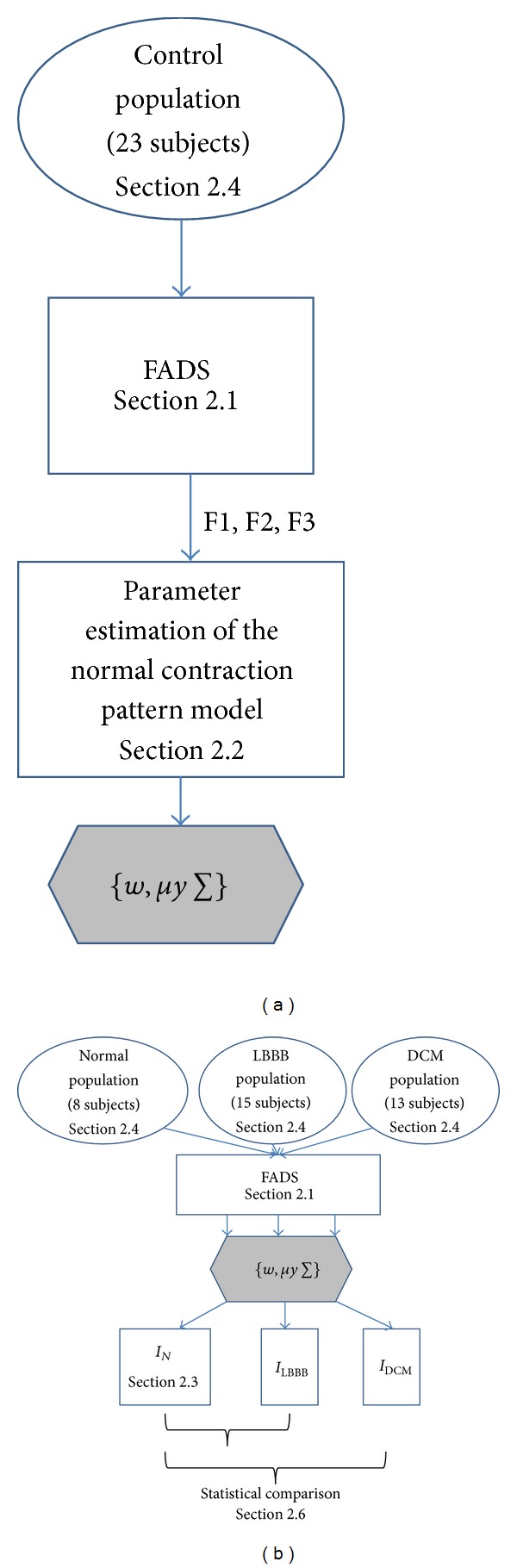
Schematic representation of the proposed methodology. (a). The normal contraction pattern was modeled as a linear combination of Gaussian density functions. The relative weights (*w*), means (*μ*) and covariance matrices (Σ) of the Gaussian mixture were estimated maximizing the mixture model likelihood of the probability density functions of the 3 most significant factors (F1, F2, and F3) computed from the Factor Analysis of Dynamic Structures (FADS) and following a standard bootstrap resampling procedure with a set of 23 control subjects. (b) Considering that the likelihood estimated on a data sample represents the probability that those observations are well described by the assumed model with *w*, *μ*, and Σ parameters, we propose indices based on this probability. A normality index (*I*_*N*_) for a group of normal subjects (not considered for the mixture parameters estimation) was measured, and statistically compared with the indices obtained for LBBB and DCM populations.

To summarize, the methodology is divided in two stages: training to obtain the model's parameters (Figure 5(a)) and application of this model to populations' comparison (Figure 5(b)).

## 3. Results

### 3.1. Factor Analysis

**Figure 6:**
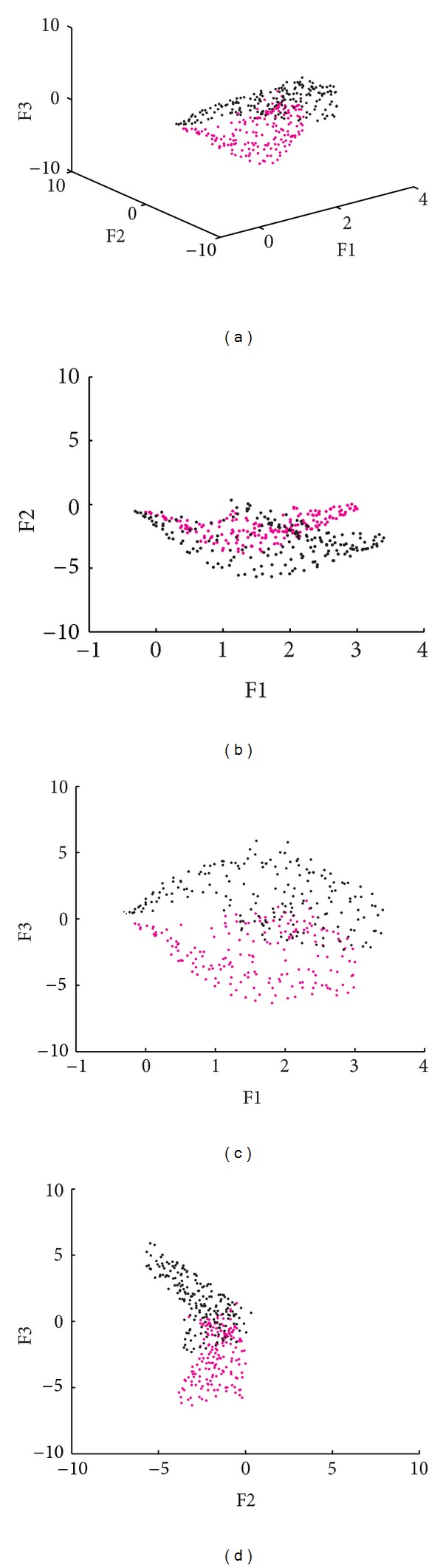
Scatter plot of the 3-MSF (F1, F2, and F3) calculated for a subject with a LBBB (right ventricle in magenta, left ventricle in black).

**Figure 7:**
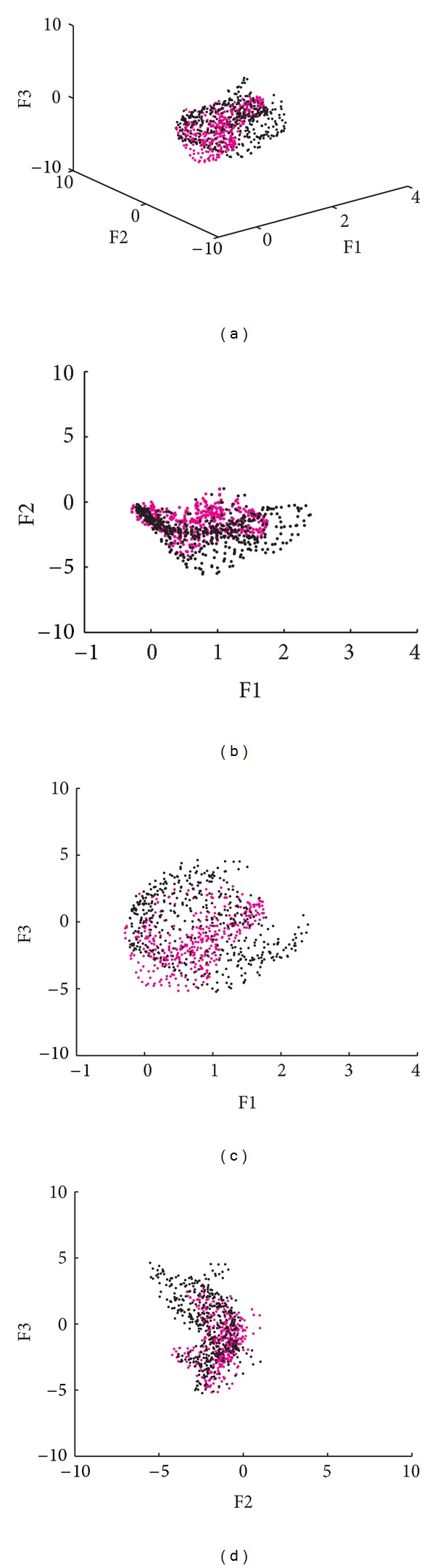
Scatter plot of the 3-MSF (F1, F2, and F3) calculated for a subject with DCM (right ventricle in magenta, left ventricle in black).

The information obtained for the 3-MSF (F1, F2, and F3) of the left and right ventricular regions was projected into scatter plots to observe differences between populations and between ventricular regions. Figures 4, 6, and 7 correspond to the Control, LBBB, and DCM populations, respectively. 

The scatter plots obtained for the subjects studied show that the information for the left and right ventricles is overlapped in the control population (Figure 4), but also in the F1 versus F2 projection for abnormal contraction patterns (Figures 6(b) and 7(b)). However, in the presence of interventricular asynchrony, as in the case of the LBBB population, it was necessary to incorporate the F3 factor information in the analysis, to appreciate a clear separation between ventricular regions (Figures 6(a), 6(c), and 6(d)). Also, for the DCM population, that presents inter- and intraventricular asynchrony, the scatter plots that incorporate the third factor information (F3) show this left and right regions partition, although it was less evident than in the case of LBBB subjects, probably explained by the difference in the asynchrony type. 

### 3.2. Model of the Factors' Probability Density Function

Different models were obtained for the PDF of the 3-MSF for six groups, with 10 subjects each, randomly selected from the control population.  Table 2 shows the characteristics of the mixture of Gaussian functions that best adjusted each group. 

For the control population, the minimum BIC and log-likelihood values corresponded to model 5, so that it has the highest probability of best describing the data corresponding to the PDF of the F1, F2, F3 distribution of the normal contraction pattern group. This model is characterized by having five Gaussian functions with variable volume, shape, and orientation. The weight parameters (*w*), mean values (*μ*), and covariance matrices (Σ) that describe the selected model are shown in Table 3. 

**Figure 8:**
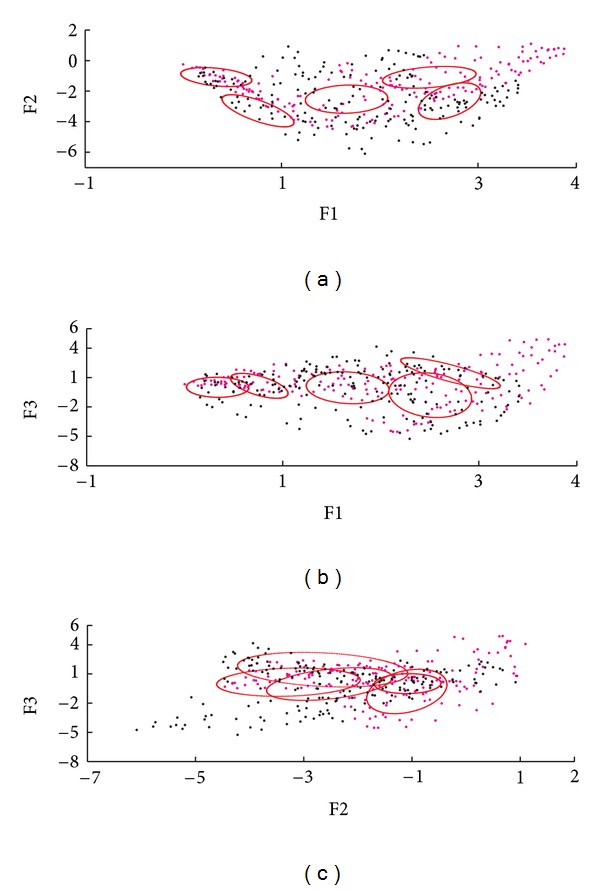
Level curves of the Gaussian functions (in red) superimposed in the dispersion plots of F1, F2, and F3 for a normal subject. Right (magenta) and left (black) ventricle regions are presented.

**Figure 9:**
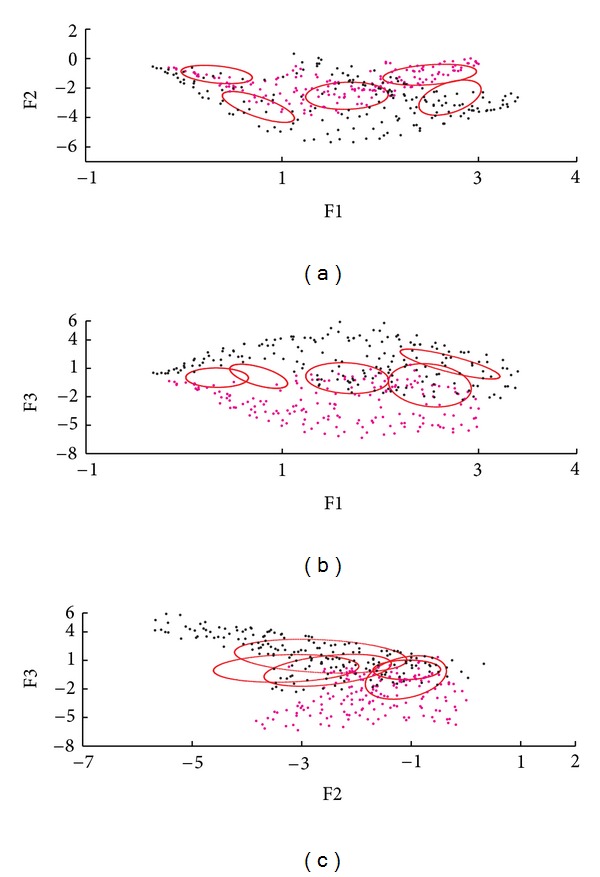
Level curves of the Gaussian functions (in red) superimposed in the dispersion plots of F1, F2, and F3 for a LBBB subject. Right (magenta) and left (black) ventricle regions are presented.

**Figure 10:**
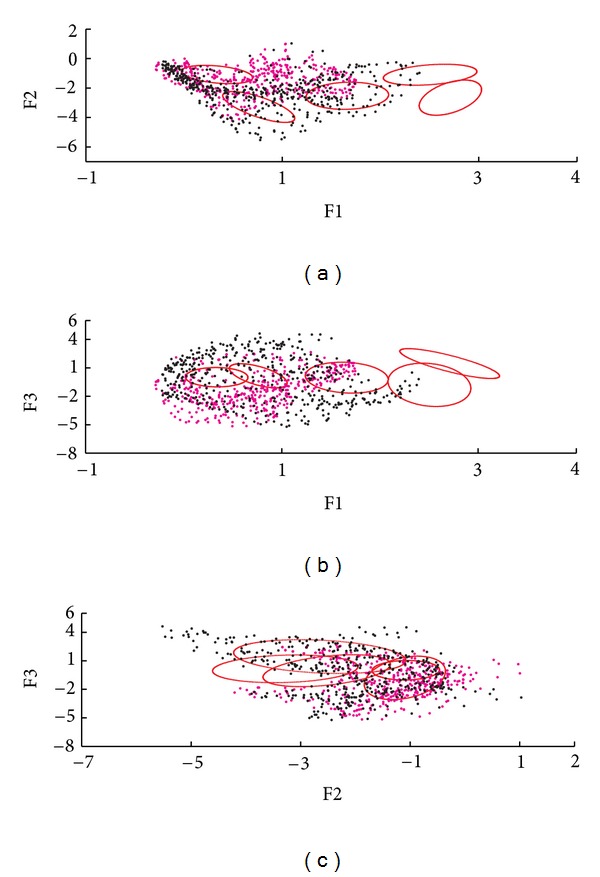
Level curves of the Gaussian functions (in red) superimposed in the dispersion plots of F1, F2, and F3 for a DCM subject. Right (magenta) and left (black) ventricle regions are presented.

The level curves of the adjusted model were superimposed with the information of the 3-MSF for one subject of each studied population. Figures 8, 9, and 10 show the correspondence for the normal, LBBB, and DCM subjects, respectively. The agreement between the model and the cardiopathic subjects is poor, as may be expected.

In Table 4, the normality indices (*I*_*N*_) obtained for the populations studied, compared with the defined normal contraction model (see (4)), are presented. It can be observed that whenever the likelihood value increases and becomes statistically different from that calculated for the normal subjects, the probability that the model explains the data decreases. The calculated indices for the pathologic populations are statistically different from the *I*_*N*_ of the normal population, which suggests that the LBBB and DCM populations present abnormalities in the ventricular contraction pattern. Additionally, the DCM population presents a larger difference compared to the reference group; this was also corroborated with the clinical characteristics of the evaluated subjects and with the deterioration of their ventricular contraction pattern.

For comparison purposes, in Table 4 the most clinically used (mean, standard deviation, and mode) indices, extracted from phase analysis, are included. It can be observed that the standard deviation obtained from the traditional analysis also shows statistical differences between the normal and pathologic populations. 

## 4. Discussion

Inter- and intraventricular contraction synchrony plays an important role in the heart pump function. The deterioration of contraction homogeneity can lead to a poor prognosis of clinical evolution, while a restoration of the ventricular contraction has proven to be of clinical benefit in patients with heart failure. However, despite the fact that several studies show that CRT can be of great benefit for severe cardiac malfunction, still up to 30% of patients do not recover after therapy. Several attempts have been made to improve patient selection and to foresee the successfulness of cardiac resynchronization therapy, depending on the particular dyssynchrony. In a large study, Chung et al. [12] concluded that echocardiographic measures of dyssynchrony are not reliable for this purpose, due to reduced sensitivity and specificity. Furthermore, a complete review by Pavlopoulos and Nihoyannopoulos [10] suggests that an overall approach must be taken to adequately select CRT candidates, where global clinical criteria, in addition to electric and mechanical dyssynchrony measures, must be considered. Van der Wall et al. [11] suggest that, despite the fact that several modalities have been proposed for the noninvasive quantification of LV dyssynchrony, there is no agreement on which technique best predicts response to CRT and that nonechocardiographic imaging techniques, such as ERNA, may provide valuable information for the selection of CRT candidates. Also, Henneman et al. [17] state that phase analysis based on SPECT can lead to an adequate detection of LV dyssynchrony and that nuclear imaging can provide valuable information for the selection of CRT candidates. In summary, the established clinical criteria to consider a subject as a CRT candidate are insufficient to identify those patients that will benefit the most from that treatment [10], justifying the need for an alternative analysis techniques, as the one presented in this work. This method considers information representative of the ventricular contraction dynamics, which is included in the three most significant factors extracted from FADS. It is based on the characterization of a normal contraction pattern, defined from a control population that is used as a reference, against which a “normality index” can be measured. The abnormality in the contraction pattern was globally measured, for two pathologic populations. 

An analysis of the 3-MSF scatter plots obtained for the populations studied indicates that the third factor information is necessary to separate left and right ventricular regions, particularly for the abnormal contraction populations, as can be observed in Figures 4, 6, and 7. These findings are in agreement with previously published results [35]. The scatter plots for the DCM patients show an increase in the data number, as well as an overlap between ventricular cavities, as compared to the LBBB population. This can be explained by the fact that DCM is characterized by an important dilation of the ventricles, together with an intrinsically heterogeneous contraction. Therefore, an increase of the information volume and cavities dispersion occurs [38]. Also, as it can be observed in Figures 8, 9, and 10, the adjustment between the normal contraction model and the cardiopathic ventricular behavior is poor, as it was expected.

The comparison of the 3-MSF PDF obtained for the normal population with respect to the behavior of LBBB and DCM groups, using the proposed index, has the advantage of not assuming a specific sequence for the normal contraction pattern and does not depend on the size of the ventricular cavities. It can be observed in Table 4 that the DCM population (inter- and intraventricular asynchrony and EF < 35%) presents a larger and more significant difference with respect to the normal population than the LBBB patients (interventricular asynchrony and normal EF). These findings are in agreement with those reported by Fauchier et al. [26], which conclude that the DCM subjects with inter- and intraventricular dyssynchrony have a greater probability of presenting an adverse cardiac event. Compared to the Fourier phase analysis, the proposed normality index is consistent with the difference obtained with the standard deviation, between normal and pathologic populations. This indicates that the normality index proposed in this work allows the evaluation of the degree of abnormality in ventricular contraction.

## 5. Conclusions

Due to the reduced sensitivity and specificity of echocardiographic measures to adequately select CRT candidates, ERNA extracted indices seem more reliable in detecting ventricular dyssynchrony. In this work, the probability density function that models a normal ventricular contraction pattern has been defined and validated for a control population. It is based on a thorough analysis of the three most significant dynamic factors obtained from ERNA images in several populations presenting different patterns of ventricular synchrony. Furthermore, a metric to quantify differences between pathologic populations and the reference normal contraction pattern has been proposed; the validation of this likelihood measure was carried out in patients with complete left bundle branch blockage and dilated cardiomyopathy. Both populations presented statistically significant differences in their contraction pattern, compared to the reference model and these differences were more important for the DCM population, due to inter- and intraventricular dyssynchrony, as expected. Compared to the Fourier phase analysis, and particularly with the standard deviation, the proposed index also detects differences between normal and pathologic groups. Furthermore, this index, together with FADS, was sensitive enough to show contraction pattern differences, which suggests that this analysis can be related to the degree of severity in the ventricular contraction dyssynchrony. Additionally, the use of this index may be promising for the followup of patients under CRT. 

In future work, other clinical information should be incorporated, either extracted from EKG or from different imaging modalities, to propose an integrated normality index, to enhance patient selection for CRT. Also, longitudinal studies through different stages of treatment will be necessary to validate the index's capacity to measure asynchrony severity, particularly in patients that have been submitted to cardiac resynchronization therapy.

## Figures and Tables

**Table 1:** Characteristics of the studied populations.

	Control	LBBB	DCM
	(*n* = 23)	(*n* = 15)	(*n* = 13)
Age (years)	28 ± 5	59.90 ± 9.09	45.6 ± 16.5
LVEF	60 ± 5.84	59.5 ± 9.4	22.2 ± 6.7
SAH *n* (%)	0	11 (73.3)	3 (20)
Diabetes mellitus *n* (%)	0	1 (6.6)	2 (13.3)
Dyslipidemia *n* (%)	0	3 (20)	4 (26.6)
Smokers *n* (%)	0	2 (13.3)	7 (46.7)

LVEF: left ventricle ejection fraction, SAH: systemic arterial hypertension, LBBB: left bundle branch block, DCM: idiopathic dilated cardiomyopathy.

**Table 2:** Characteristics of the models that best describe the contraction pattern for the six defined groups.

Model	Type	Number of components	BIC	Log-likelihood
1	VVV	5	34258.15	16928.97
2	VVV	5	34094.83	16847.28
3	VEV	8	34502.55	16984.84
4	VEV	6	33676.04	16597.08
5	VVV	5	32863.10	16232.13
6	VEV	8	34502.55	16984.84

BIC: bayes Information criterion, Log-likelihood: logarithm of the likelihood value, VEV: Ellipsoidal, same shape, variable orientation; VVV: Ellipsoidal, varying volume, shape, and orientation.

**Table 3:** Weight parameters (*w*), mean values (*μ*), and covariances (Σ) for the model that best describes the PDF of the 3-MSF of the control population.

Function	Weight (*w*)	Mean (*μ*)	Covariance (Σ)
			
*N*_1_	0.154	$\left[\begin{array}{c} 0.336\\ -1.080\\ 0.010 \end{array}\right]$	$\left[\begin{array}{ccc} 0.131 & -0.077 & -0.003\\ -0.077 & 0.387 & 0.027\\ -0.003 & 0.027 & 1.047 \end{array}\right]$

			
*N*_2_	0.443	$\left[\begin{array}{c} 1.661\\ -2.524\\ -0.036 \end{array}\right]$	$\left[\begin{array}{ccc} 0.179 & -0.010 & -0.108\\ -0.011 & 1.352 & 0.788\\ -0.101 & 0.788 & 2.818 \end{array}\right]$

			
*N*_3_	0.139	$\left[\begin{array}{c} 2.504\\ -1.096\\ -0.794 \end{array}\right]$	$\left[\begin{array}{ccc} 0.231 & 0.087 & -0.168\\ 0.087 & 0.567 & 0.516\\ -0.168 & 0.516 & 5.219 \end{array}\right]$

			
*N*_4_	0.169	$\left[\begin{array}{c} 0.759\\ -3.283\\ 0.160 \end{array}\right]$	$\left[\begin{array}{ccc} 0.165 & -0.425 & -0.026\\ -0.425 & 1.763 & 0.290\\ -0.026 & 0.290 & 2.120 \end{array}\right]$

			
*N*_5_	0.094	$\left[\begin{array}{c} 2.710\\ -2.652\\ 1.448 \end{array}\right]$	$\left[\begin{array}{ccc} 0.283 & 0.612 & -0.533\\ 0.612 & 2.479 & -0.641\\ -0.533 & -0.641 & 3.157 \end{array}\right]$

**Table 4:** Normality indices (see (4)) calculated for Normal, LBBB, and DCM subjects.

	Normal	LBBB	DCM
	(*n* = 8)	(*n* = 15)	(*n* = 15)
FADS			

*I*_*N*_	1.17 ± 0.12	1.55 ± 0.05*	1.70 ± 0.07*

Phase analysis			

Mean	127.22	154.13*	156.12
(min, max)	(117.51, 132.63)	(141.79, 170.63)	(128.94, 190.11)
Std. Dev.	12.72	19.36*	46.3405*
(min, max)	(11.19, 14.25)	(18.27, 23.41)	(34.35, 61.24)
Mode	126	150*	156
(min, max)	(118.50, 130.50)	(138, 180)	(132, 198)

*P < 0.01 with respect to the control group.
